# Supplementary material for: The impact of experimental design choices on parameter inference for models of growing cell colonies
Source: R Soc Open Sci. 2018 Aug 29;5(8):180384. doi: 10.1098/rsos.180384 (PMC6124093; doi:10.1098/rsos.180384)
Supplement: Supplementary Material [file rsos180384supp1.pdf]

Supplementary Material

The impact of experimental design choices on parameter inference for models of growing cell colonies

Andrew Parker[a], Matthew J. Simpson[b], Ruth E. Baker[a,*]

[a] *Mathematical Institute, University of Oxford, Oxford, UK.*
[b] *School of Mathematical Sciences, Queensland University of Technology, Brisbane, Australia.*

Abstract

In this Supplementary Material document we present additional evidence that demonstrate consistency of our results in the main report across different parameter regimes, different experimental conditions, and an extended simulation domain.

1. Results for different parameter regimes

In the main text, synthetic data are generated with $P_m = 0.25$ and $P_p = 0.0025$, giving $Ratio := P_m/P_p = 100$. To test the consistency of our results across a range of cell types, we present additional results here from identical simulations as in the main text with $Ratio = 10$ or $Ratio = 1$. As the 3T3 fibroblast and MDA MB 231 cell lines we consider in the main text have similar proliferation rates we keep P_p fixed and vary P_m to $P_m = 0.025$ or $P_m = 0.25$, corresponding to $Ratio = 10$ or $Ratio = 1$. Our results for these new parameter regimes are shown in Supplementary Figures S1, S2, S3 and S4. The trends in these results are consistent with those in the main text. Note that, as the parameter ratio, $Ratio$, decreases (through decreasing P_m), we obtain less information from the trajectory-based statistics, $\|x\|$ and Γ, as we might anticipate. Further, the information gained from the pairwise correlations statistics, C_{XY} and C_Y, also decreases as $Ratio$ is reduced, but only when we initialise cells over six rows.

2. Results for longer experiments

In the main text, we performed simulations over 12 h, as most experimental data of cell spreading is observed for at least this amount of time. Here we investigate how our results change if we have

*Corresponding author, `ruth.baker@maths.ox.ac.uk`

data from a longer experiment. In Figure S5 we consider the change in the information obtained from ABC parameter inference in the case where simulations and experiments are performed over 24 or 36 h, using the same parameter values as in the main document ($P_m = 0.25$, $P_p = 0.0025$). For each statistic considered, the information gained is either the same or slightly higher when simulating for 24 h rather than 12 h. For some statistics, more information still is gained when simulating for 36 h instead of 12 h (e.g. the connectivity statistics, κ_4 and κ_8), however for others there is a drop in the information gained (e.g. the quadrat statistics, Q_2 and Q_4).

3. Results incorporating a larger spatial domain

Instead of simulating our model on a domain corresponding to that of the experimental image, we can more accurately replicate the experimental domain by initialising cells and simulating in a larger $R = 72 \times C = 96$ grid, and compute statistics only by considering cells in the central portion of that domain, of size $R = 24 \times C = 32$, as performed previously. By considering a snapshot of a larger domain, we are accounting for situations when cells may move into or out of the part of the domain under observation, which may influence the resulting statistics. The domain configurations are demonstrated in Figure S6 (a), and the difference in information obtained from ABC parameter inference for each statistic when initialising cells over different number of rows in the domain are shown in Figure S6 (b). The information gain from each statistic is very much consistent with the information gains shown in the main report.

Data accessibility. Code used to generate the data are available from `https://github.com/andrew-parker/Impact-of-experimental-design`.

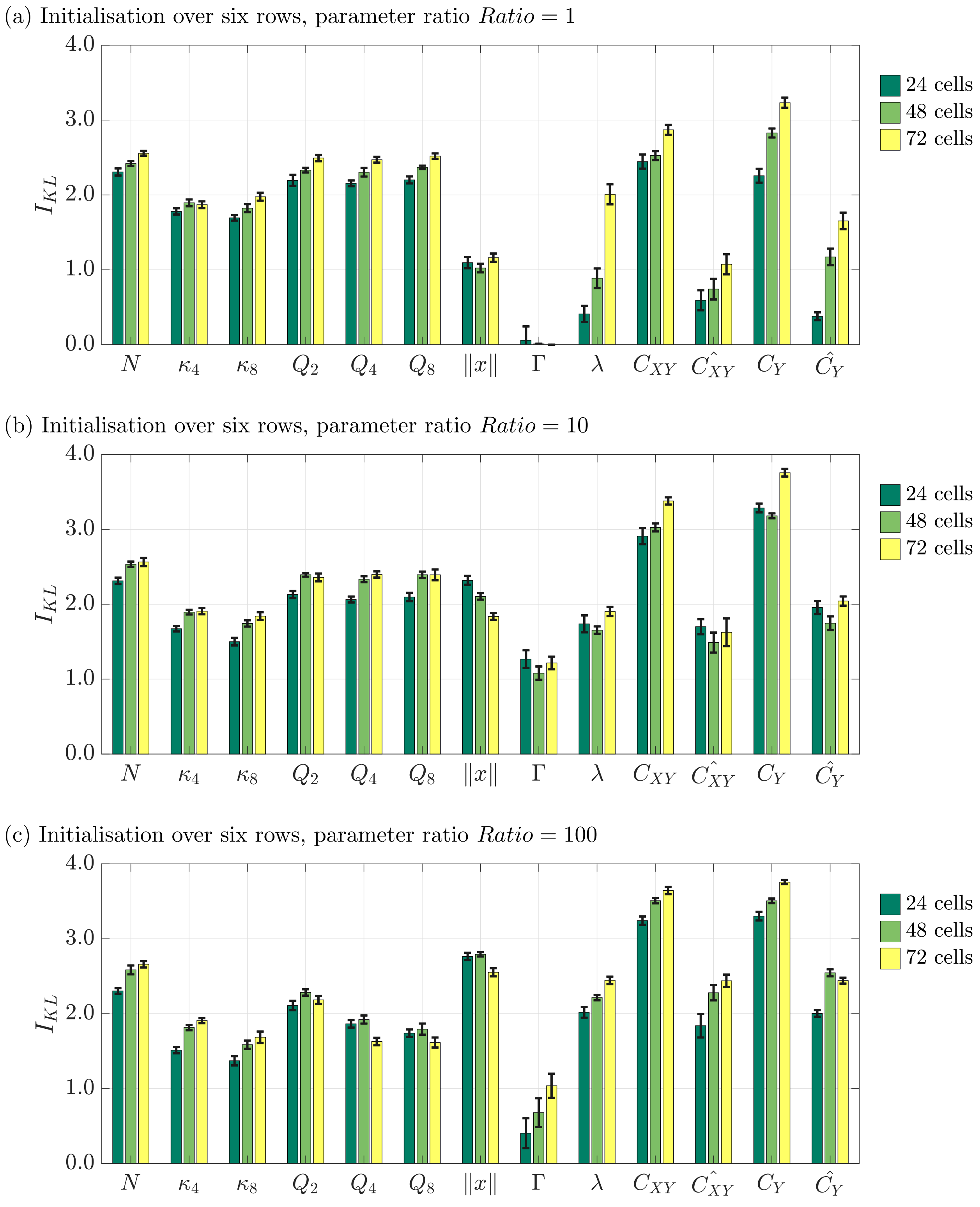

Figure S1: Information gain in moving from the prior to the posterior for each summary statistic when initialising a scratch assay with 24, 48, or 72 cells. (a) Experimental statistics are computed with $Ratio = 1$ corresponding to $P_m = 0.0025$, $P_p = 0.0025$. (b) Experimental statistics are computed with $Ratio = 10$, corresponding to $P_m = 0.025$, $P_p = 0.0025$. (c) Experimental statistics are computed with $Ratio = 100$, corresponding to $P_m = 0.25$, $P_p = 0.0025$.

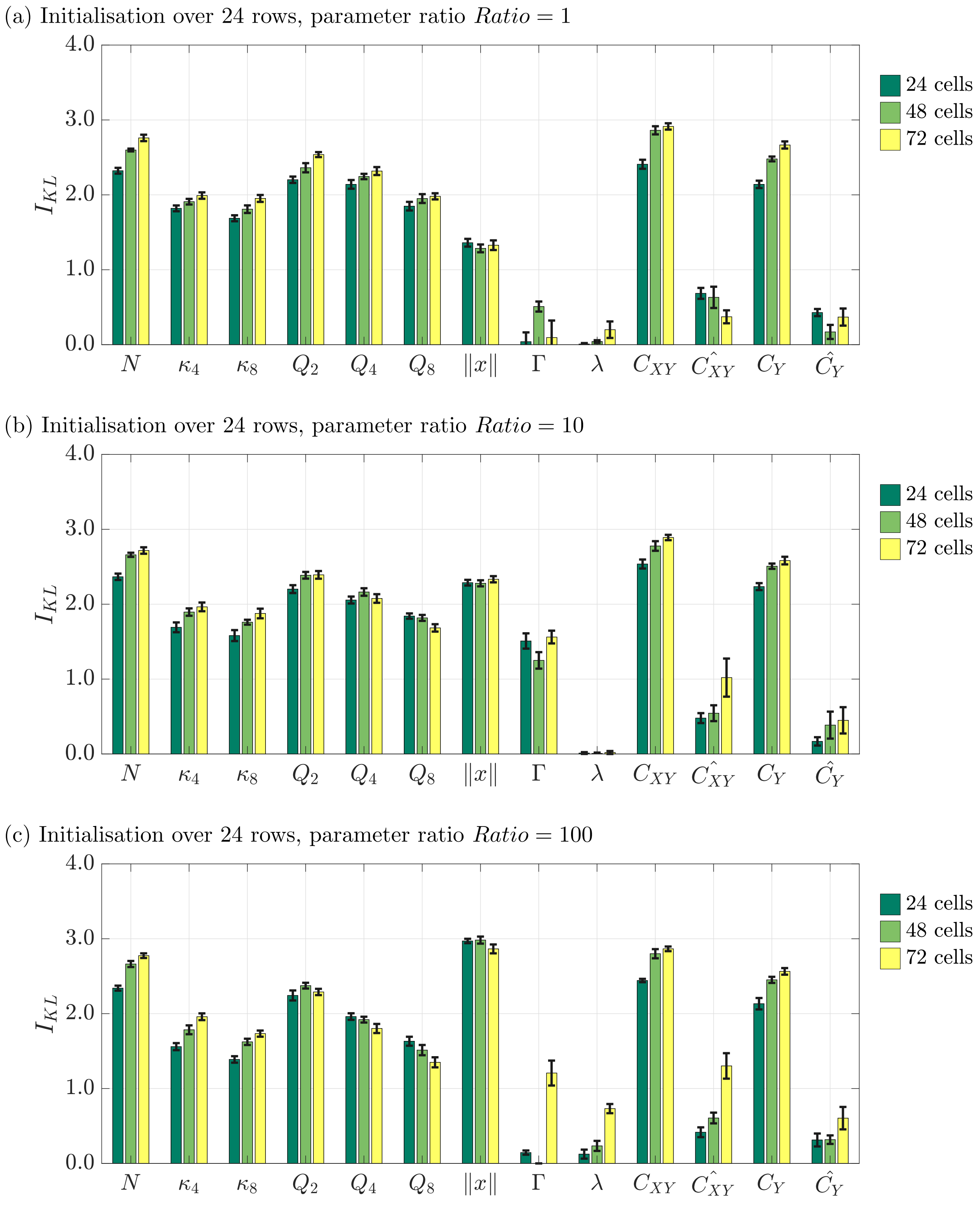

Figure S2: Information gain in moving from the prior to the posterior for each summary statistic when initialising a growth-to-confluence assay with 24, 48, or 72 cells. (a) Experimental statistics are computed with $Ratio = 1$ corresponding to $P_m = 0.0025$, $P_p = 0.0025$. (b) Experimental statistics are computed with $Ratio = 10$, corresponding to $P_m = 0.025$, $P_p = 0.0025$. (c) Experimental statistics are computed with $Ratio = 100$, corresponding to $P_m = 0.25$, $P_p = 0.0025$.

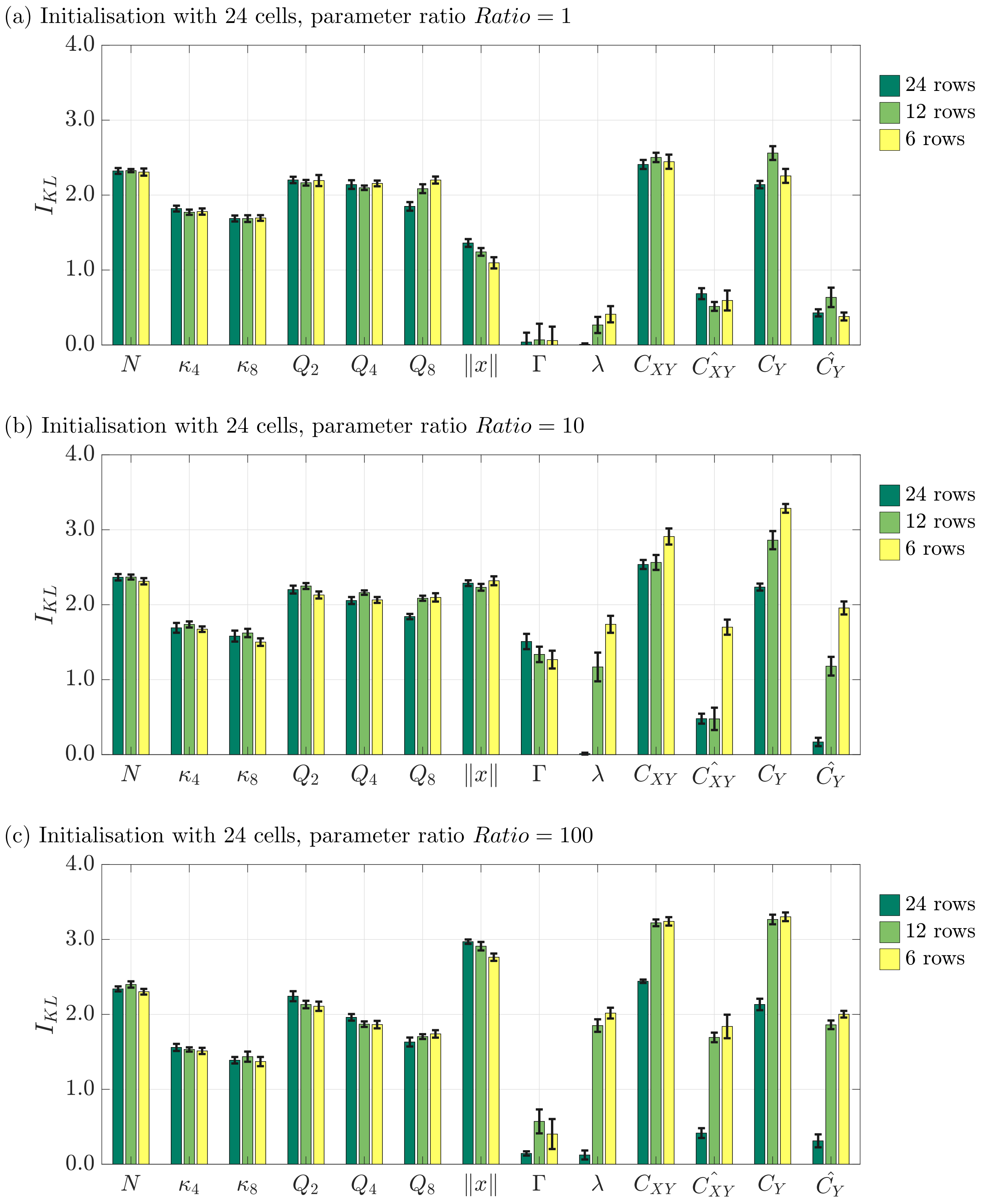

Figure S3: Information gain in moving from the prior to the posterior for each summary statistic and for each experimental design when initialised with 24 cells. (a) Experimental statistics are computed with $Ratio = 1$ corresponding to $P_m = 0.0025$, $P_p = 0.0025$. (b) Experimental statistics are computed with $Ratio = 10$, corresponding to $P_m = 0.025$, $P_p = 0.0025$. (c) Experimental statistics are computed with $Ratio = 100$, corresponding to $P_m = 0.25$, $P_p = 0.0025$.

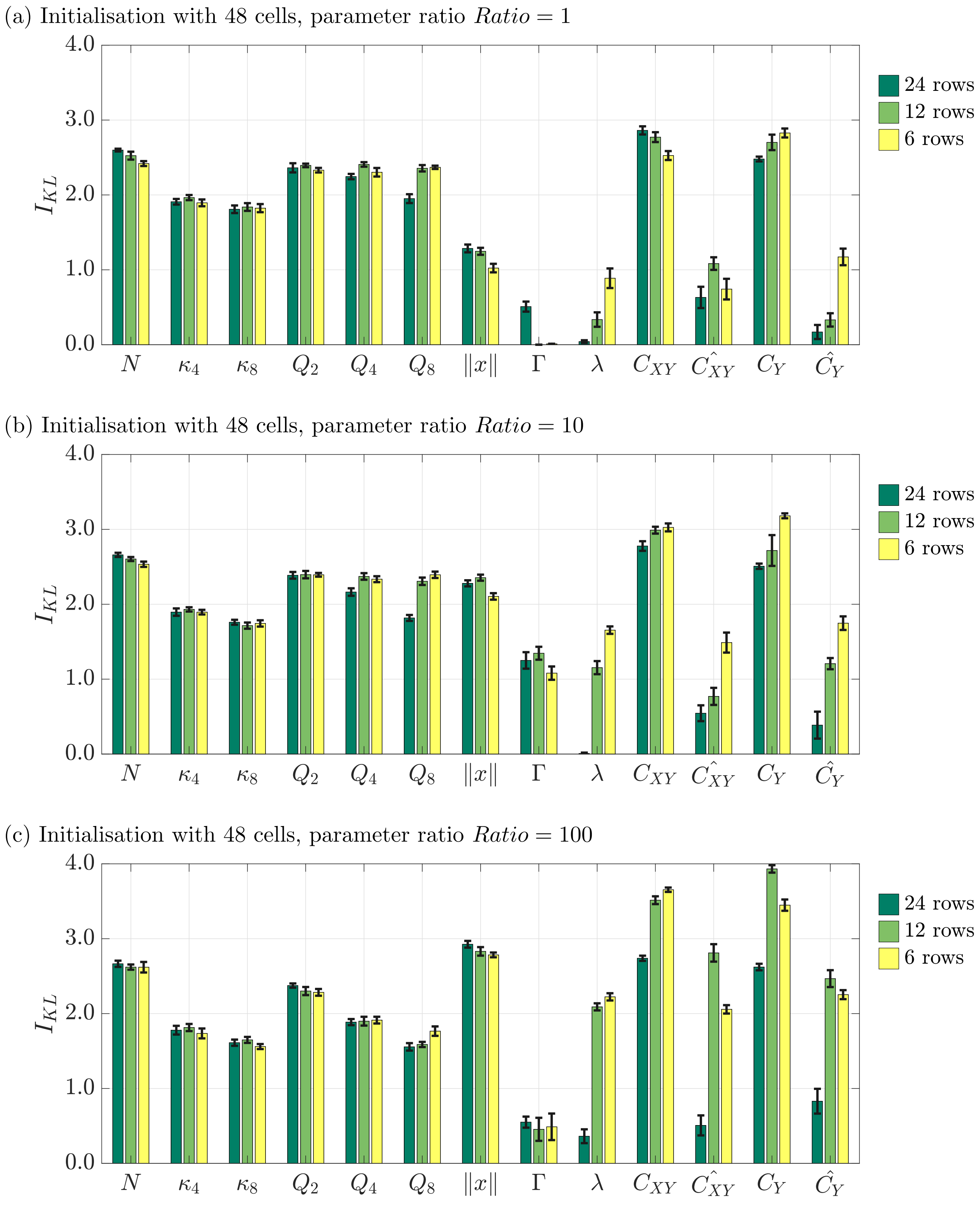

Figure S4: Information gain in moving from the prior to the posterior for each summary statistic and for each experimental design when initialised with 48 cells. (a) Experimental statistics are computed with $Ratio = 1$ corresponding to $P_m = 0.0025$, $P_p = 0.0025$. (b) Experimental statistics are computed with $Ratio = 10$, corresponding to $P_m = 0.025$, $P_p = 0.0025$. (c) Experimental statistics are computed with $Ratio = 100$, corresponding to $P_m = 0.25$, $P_p = 0.0025$.

(a) Initialisation with 24 cells, simulated for 36 hours, parameter ratio $Ratio = 100$

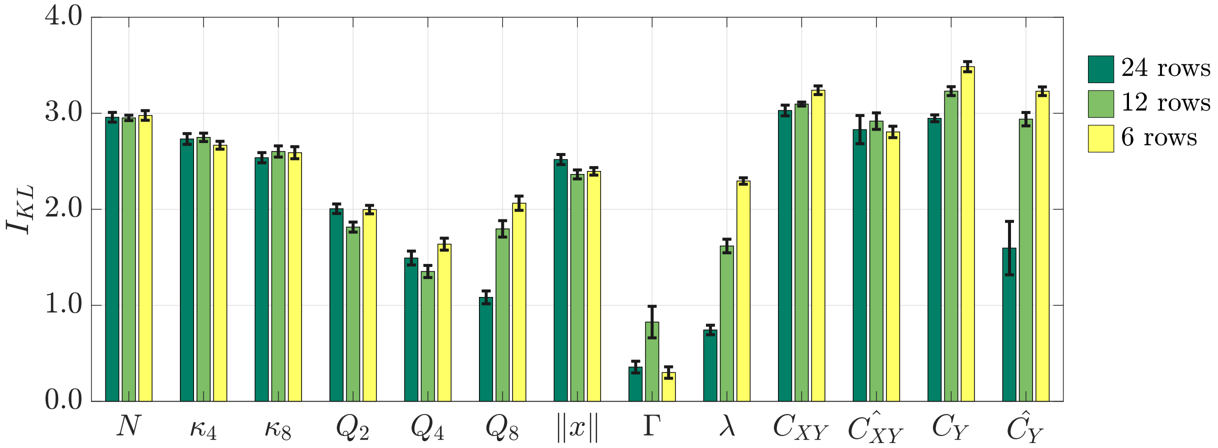

(b) Initialisation with 24 cells, simulated for 24 hours, parameter ratio $Ratio = 100$

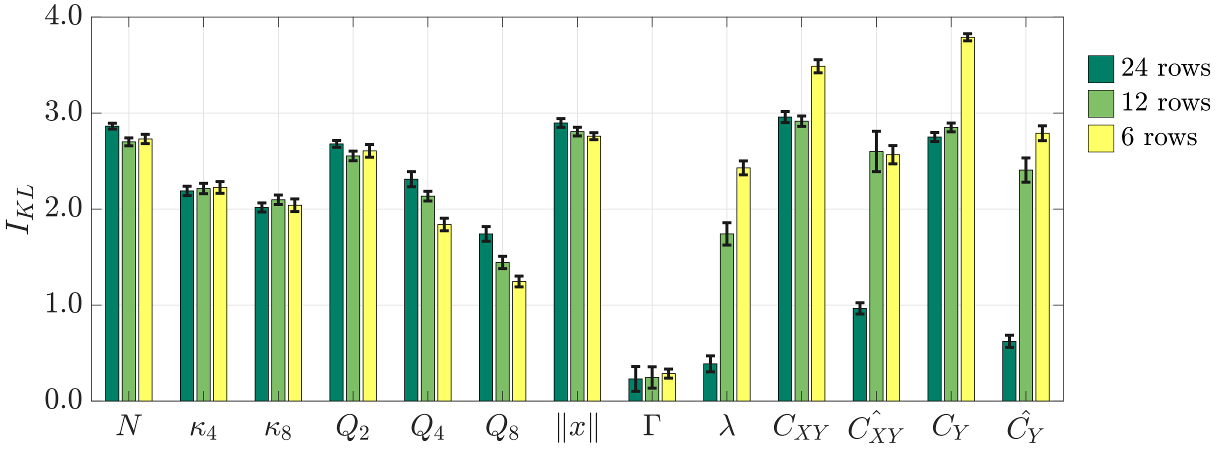

(c) Initialisation with 24 cells, simulated for 12 hours, parameter ratio $Ratio = 100$

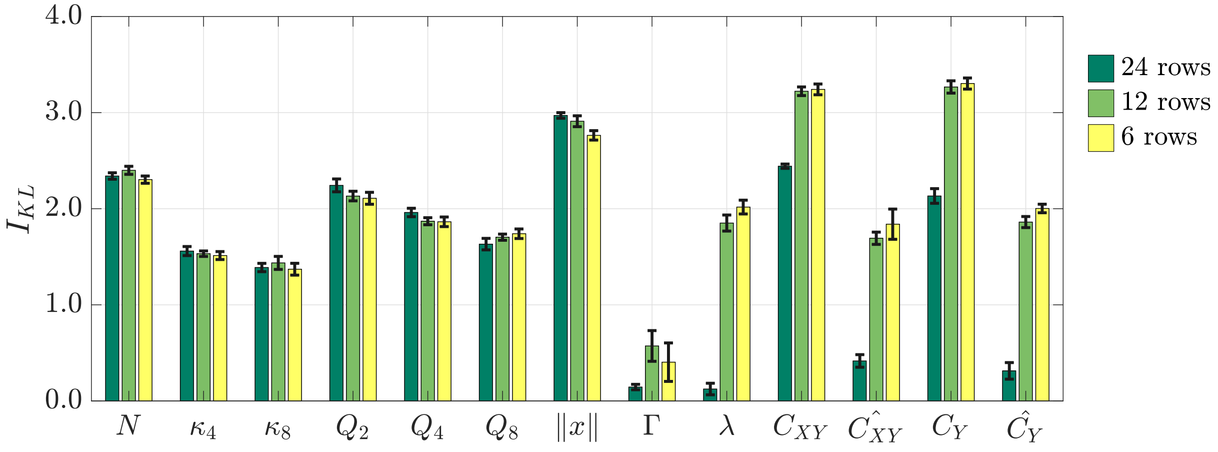

Figure S5: Information gain in moving from the prior to the posterior for each summary statistic and when initialised with 24 cells and the length of the simulations are varied. Experimental statistics are computed with $Ratio = 100$, corresponding to $P_m = 0.25$, $P_p = 0.0025$. (a) Simulations are run for 36 hours, corresponding to three times the length of the simulations in the main report. (b) Simulations are run for 24 hours. (c) Simulations are run for 12 hours.

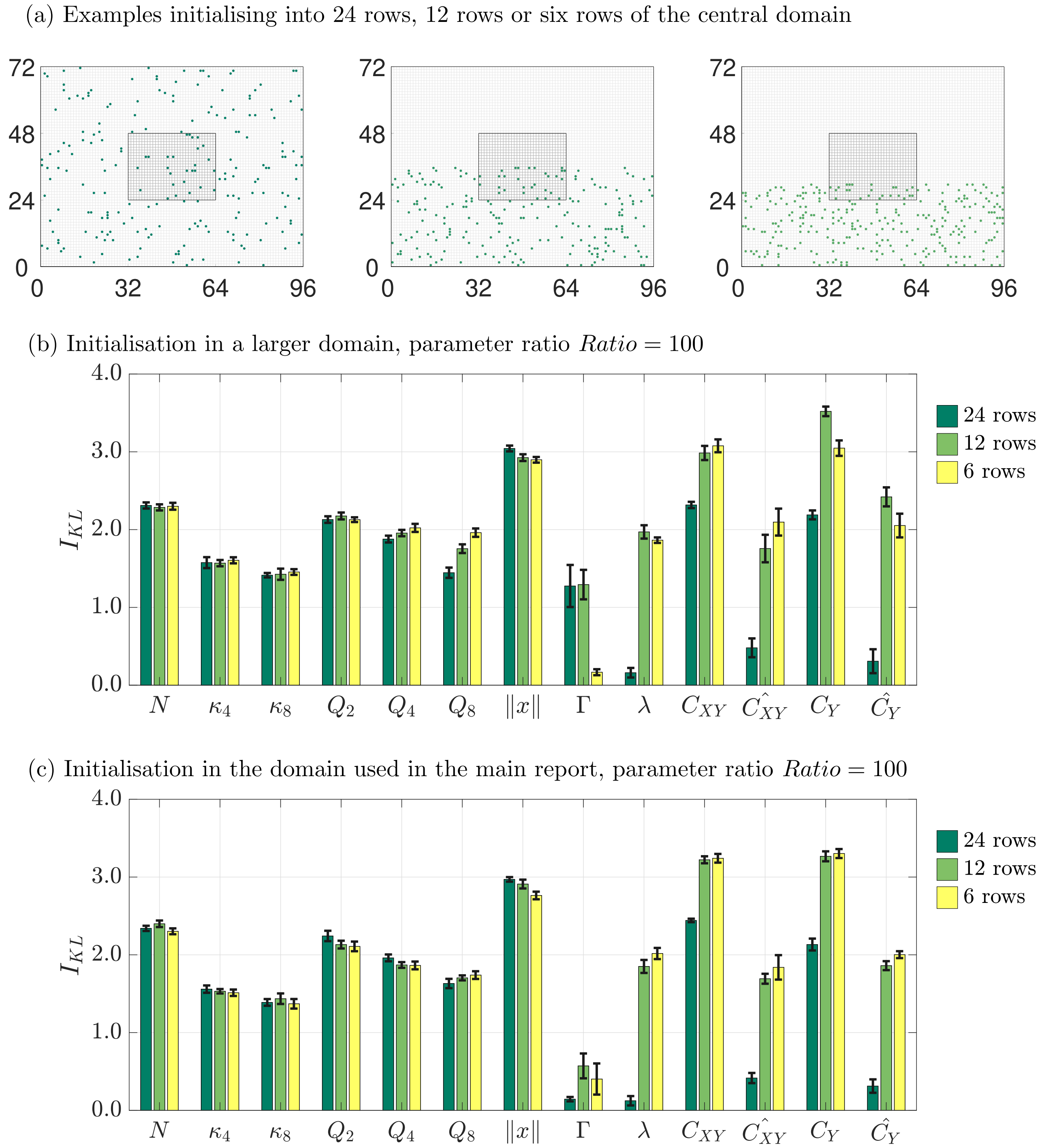

Figure S6: Information gain in moving from the prior to the posterior for each summary statistic when initialised with a larger domain ($R = 72$, $C = 96$), and computing statistics using only the central portion of the domain. (a) Example experimental domains, corresponding to a growth-to-confluence assays (24 rows) or scratch assays over either half the central domain (12 rows) or one quarter of the central domain (6 rows). (b) Experimental statistics are computed with $Ratio = 100$, corresponding to $P_m = 0.25$, $P_p = 0.0025$. (c) The version of (b) from the main report.
